# User Experience, Satisfaction, and Complications of Direct-to-Consumer Orthodontics in Spain: A Cross-Sectional Study

**DOI:** 10.3390/jcm14072382

**Published:** 2025-03-30

**Authors:** Milagros Adobes Martin, Adriana Pérez Márquez, Simonetta Meuli, Adrian Curtó Aguilera, Mario Dioguardi, Riccardo Aiuto, Daniele Garcovich

**Affiliations:** 1 Department of Dentistry, Universidad Europea de Valencia, 46010 Valencia, Spain; milagros.adobes@universidadeuropea.es (M.A.M.); 22124708@live.uem.es (A.P.M.); 2 Department of Orthodontics, Universitá Cattolica del Sacro Cuore, 00168 Rome, Italy; simonetta.meuli@unicatt.it; 3 Department of Surgery, Universidad de Salamanca, 37007 Salamanca, Spain; adrian_odonto@usal.es; 4 Department of Clinical and Experimental Medicine, University of Foggia, 71100 Foggia, Italy; mario.dioguardi@unifg.it; 5 Department of Biomedical, Surgical, and Dental Science, University of Milan, 20122 Milan, Italy; riccardo.aiuto@unimi.it

**Keywords:** corrective orthodontics, clear aligners, patient satisfaction, treatment outcome

## Abstract

**Background/Objectives:** Direct-to-Consumer Orthodontics (DTCO) has gained popularity through social media, offering clear aligner treatments without in-person supervision. However, there is limited research on the related user experiences, satisfaction, and complications, particularly in Spain. This study aimed to evaluate user experiences, satisfaction levels, and complications associated with DTCO in a sample of Spanish consumers. Additionally, differences among major DTCO providers were analyzed. **Methods:** A cross-sectional online survey was conducted over one year. This questionnaire, adapted from previous studies and reviewed by certified orthodontists, assessed user awareness, reasons for treatment selection, communication with providers, discomfort, complications, and satisfaction. A total of 101 valid responses were analyzed using statistical means. **Results:** The majority of respondents reported that their expectations were not met, and they would not recommend DTCO. Cost was the primary motivation for treatment selection, followed by convenience. Many users experienced complications, leading to dental consultations. While overall satisfaction levels did not significantly differ among providers, variations were observed in discomfort levels, in-person care availability, and the need for additional consultations. **Conclusions:** DTCO offers affordability and convenience but raises concerns regarding treatment quality and supervision. In most cases, users’ expectations were not met, highlighting the need for greater patient education and regulatory oversight.

## 1. Introduction

In recent decades, orthodontics has undergone a significant transformation due to technological advances and the increasing accessibility of treatment options, particularly Direct-to-Consumer Orthodontics (DTCO) and the influence of social media. Since 2020, DTCO has gained popularity among those seeking more affordable and convenient alternatives for the correction of dental alignment without requiring a professional diagnostic examination, X-rays, or in-person follow-up [1].

DTCO includes two approaches: The first is “Do-It-Yourself” (DIY) orthodontics, where the consumer receives materials at home to take dental impressions and sends them back to the company by mail. The second involves intraoral scanners, requiring consumers to visit a dental office or facility where intraoral scan records can be obtained. In both cases, a remote technician or dentist designs the treatment plan and creates the aligners. Once the aligners are received, users submit photos weekly or biweekly via a mobile app for remote evaluation or monitoring [2]. This phenomenon has sparked debates in the dental profession and society regarding the quality, safety, and effectiveness of these treatments.

The literature on DTCO emerged in 2017 with an article published by the American Association of Orthodontists (AAO) titled “Orthodontics Report Uptick in Number of Patients Attempting DIY Teeth Straightening”. The article revealed that 13% of patients attempted DIY orthodontic practices, such as using adhesive tape to close gaps, pushing teeth with fingers, or using pencils to apply pressure. These actions led to complications such as dental mobility, periodontitis, and occlusal instability.

A 2020 survey of 270 orthodontists across the U.S. and Canada found that four out of ten reported a decline in patient numbers due to this type of orthodontic device [3]. Many orthodontists argue that DTCO’s lower cost is attributed to reduced material quality and the absence of in-person supervision [2,3]. Additionally, by eliminating the orthodontist, companies can provide a more cost-effective service to consumers [4].

While DTCO expands access to orthodontic care, concerns have been raised regarding the quality, safety, and effectiveness of these treatments. The lack of in-person supervision has been associated with undiagnosed dental conditions; complications such as malocclusion instability, gum inflammation, and pain; and an increased reliance on traditional dentists for corrective interventions. In 2020, the American Association of Orthodontists (AAO) publicly warned against moving teeth without professional supervision, issuing a consumer alert and filing legal actions with 36 state dental boards against SmileDirectClub^®^, the pioneering Direct-to-Consumer Orthodontics company in the U.S. [5,6].

DTCO is characterized by remote supervision, the influence of social media, and aggressive marketing strategies [1,6]. The advertising strategies used play a key role in the transition of orthodontic approaches [7]. These marketing techniques present a visually appealing and simplified message to attract uninformed customers. They emphasize the benefits and process while often omitting information on potential adverse effects, alternative treatments, or complementary techniques necessary for optimal results [8].

Social media platforms such as Instagram, Facebook, Twitter, and TikTok play an essential role in spreading information and opinions about orthodontics, shaping public perception and influencing decision making. The use of hashtags allows users to search for specific content, with posts listed in order of relevance or chronology [9,10].

Influencer and celebrity marketing has become a powerful tool for DTCO companies. Influential users promote these products to their followers, sharing personal experiences, highlighting advantages, and explaining the need for treatment.

To date, little research has explored the health impacts of DTCO. Apart from media reports, limited information is available on the risks or benefits experienced by users. There is a gap in the relevant literature regarding who these consumers are, why they choose DTCO, whether they consult dentists before starting treatment, and if they are satisfied. Understanding this information could help to clarify the social phenomenon of DTCO, predict its trajectory, and improve professional interactions with patients.

Existing studies on DTCO have focused primarily on patient perceptions in the United States and Canada, with limited data from European populations [5,6]. The Spanish market for DTCO is expanding, yet there is no published research evaluating user experiences, satisfaction, and complications in Spain. This study aims to fill this gap by analyzing how Spanish consumers perceive DTCO, their satisfaction levels, and the challenges that they encounter.

The primary aim of this study was to evaluate, in a sample of the Spanish population, the level of satisfaction of users who have undergone or are currently undergoing treatment with Direct-to-Consumer Orthodontics, and whether a difference exists based on the chosen company.

Moreover, we aimed to examine differences in the online information provided by the two leading DTCO companies in Spain, Impress^®^ and DrSmile^®^, as well as a pioneering U.S. company, SmileDirectClub^®^. This analysis included expectations, satisfaction, aligner fit, discomfort, treatment recommendation, and availability. Additionally, this study assessed participant awareness based on their brief responses regarding current issues with Direct-to-Consumer Orthodontics, their perceptions of the available information, and their treatment outcomes.

## 2. Materials and Methods

This observational and cross-sectional study was conducted by two operators using an online survey between December 2023 and December 2024. The online survey was designed using Google Forms (Google LLC, Mountain View, CA, USA) and consisted of 30 items which were based on those used in the study by Anna Wexler et al., “Direct-to-consumer Orthodontics: Surveying User Experience” [5]. A survey review process was conducted to ensure methodological rigor while maintaining comparability with the validated questionnaire used by Wexler et al. (2020) [5]. A prior literature review was performed to refine and modify relevant questions, enhancing the original questionnaire’s comprehensiveness. The revised version was evaluated by two experienced and certified orthodontists, who assessed its ability to capture key aspects of user experience, including how individuals learned about Direct-to-Consumer Orthodontics (DTCO), their motivations for choosing this treatment, interactions with dental professionals before and during treatment, perceived complications, levels of discomfort, communication with the provider, and sociodemographic characteristics.

To ensure linguistic and cultural accuracy, the survey was first translated into Spanish by a professional bilingual translator and then back-translated into English by an independent translator who was unfamiliar with the original questionnaire. Discrepancies between the original and back-translated versions were resolved by consensus of two orthodontic specialists, in order to maintain conceptual equivalence while adapting the content for Spanish respondents. While the total number of questions remained unchanged, modifications were made to improve the questionnaire’s ability to capture user experiences specific to the Spanish population. Instead of adding new questions, response options were expanded to provide more detailed insights into factors such as complications, professional consultations, and the influence of social media. Additionally, all demographic questions were adapted to a European perspective, ensuring relevance to the Spanish context. The insurance-related questions were also modified, as health insurance coverage for orthodontic treatments is less common in Spain compared to the United States, requiring adjustments to better reflect the realities of the Spanish healthcare system. This approach ensured that the study maintained the opportunity for direct comparison with Wexler et al.’s findings while allowing for a more nuanced understanding of the experiences of Spanish DTCO users. Minor wording refinements ensured cultural relevance while preserving the original survey’s intent. These adaptations strengthened the questionnaire’s validity, ensuring that it remained methodologically sound, linguistically precise, and applicable to the Spanish DTCO population. The questions are displayed in Table 1.

This study did not require approval from the Ethics Committee of the European University of Madrid, as the survey was completely anonymous and did not collect personally identifiable information. Participation was voluntary, non-invasive, and posed no risk to participants. The study only gathered general opinions, perceptions, and user experiences related to Direct-to-Consumer Orthodontics, without addressing sensitive topics such as medical history or psychological well-being. Additionally, the research complied with national data protection laws, including the General Data Protection Regulation (GDPR) in Europe and the Spanish Organic Law 3/2018 on Personal Data Protection and Digital Rights. This confirmation aligns with ethical standards for anonymous online survey research, where formal ethical approval is typically waived under these conditions. This study used a convenience sampling strategy, recruiting participants based on their engagement with DTCO-related content on Facebook and Instagram. Convenience sampling allowed for targeted recruitment of individuals actively discussing or considering DTCO treatment, providing valuable insights. However, this method may not fully represent the entire Spanish DTCO user population, particularly those who are less active on social media or who did not interact with DTCO-related content. All participants provided informed consent before completing the survey. Respondents were recruited based on the following inclusion criteria: (a) users who had been in treatment for at least two months or had previously undergone treatment with direct-to-consumer aligners, (b) possessed the visual capacity to complete the survey, and (c) had access to mobile technology and an internet connection. Exclusion criteria included the following: (a) users below the age of 18, (b) individuals posting in a language other than Spanish, (c) users posting on behalf of commercial entities, (d) posts that were financially motivated or sponsored by a company, (e) users who promoted the aligners without having used them, and (f) users with less than two months of treatment. The survey was distributed to 517 social media profiles, obtaining 102 responses. One response was discarded due to withdrawal of consent or incomplete answers, leaving 101 valid responses. Participants were recruited based on their interaction or feedback with social media posts (comments and/or likes) on the Facebook and Instagram accounts of the three included DTCO companies: Impress^®^, DrSmile^®^, and SmileDirectClub^®^. Users who engaged with content related to DTCO—such as promotional posts, customer experiences, or treatment discussions—were considered for participation. If the user met the inclusion criteria, a private message was sent to their personal account from one of the operators’ Facebook or Instagram accounts with a link to the survey, along with a brief explanation of the study’s objective. After completing the survey, users had the opportunity to enter a raffle to win one of three EUR 30 Spotify Premium gift cards. The statistical analysis was conducted using the SPSS 23 software (IBM Corp., Armonk, NY, USA) with a confidence level of 95%. An a priori sample size calculation using the G*Power software (Version 3.1.9.7) (Heinrich-Heine-Universität Düsseldorf, Düsseldorf, Germany) determined that a minimum of 88 participants was needed to detect significant differences, considering an expected effect size of 0.3, a power of 80%, and an alpha level of 0.05. These parameters were chosen following Cohen’s conventional effect size benchmarks for social and medical research, where 0.3 represents a moderate effect which is appropriate for studies analyzing categorical data in a non-clinical setting [11]. To account for potential dropouts and ensure statistical power, the final sample size was set to be approximately 100 respondents. Chi-square tests were performed to analyze differences in questionnaire responses between user groups, with results considered statistically significant for *p*-values below 0.05. To analyze potential differences in questionnaire responses between users of different companies, Chi-square tests were performed. Open-ended answers, if available, were analyzed using thematic analysis.

## 3. Results

The survey was sent to 517 social media profiles and 102 responses were obtained, of which 1 was discarded due to the withdrawal of consent. Therefore, the final sample consisted of 101 respondents.

### 3.1. Sociodemographic Characteristics

Of the 101 participants, 74.26% were female and 25.74% male. Most respondents belonged to the age range of 31–43 years. Regarding marital status, 35.64% identified as single, 34.65% as in a relationship, and 25.74% as married. In terms of educational level, 32.67% held a master’s degree, 25.74% a university degree, and 22.77% a higher education diploma. The majority (58.42%) were employed full-time (Table 2).

### 3.2. Channels of Treatment Discovery

**Figure 1:**
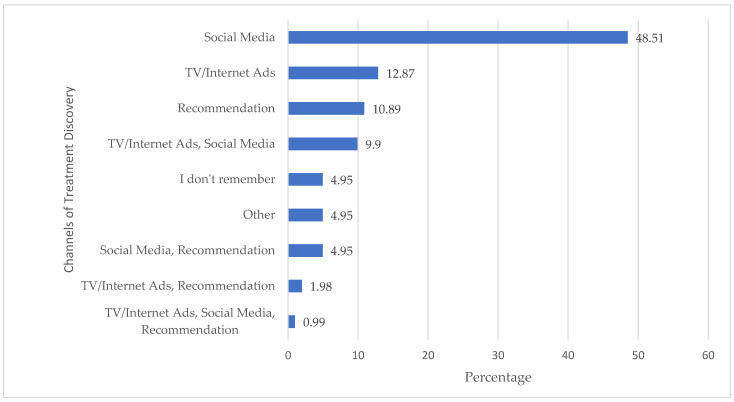
Horizontal bar chart displaying the percentage of respondents (*x*-axis) who discovered the treatment through different information channels (*y*-axis); the question allowed for the selection of multiple options.

The question used to explore the channels for treatment discovery allowed multiple options to be selected. As reported in Figure 1, social media platforms were the primary source for discovering DTCO treatments, being selected by 64% of respondents; either individually or combined with other channels such as TV/Internet advertisements and recommendations from acquaintances.

### 3.3. Reasons for Treatment and Company Selection

The most frequent motivations for opting for direct-to-consumer aligner treatment were dental crowding (66.34%), bite improvement (27.72%), and interdental gaps (13.86%). Impress^®^ was the most frequently chosen company (47.52%), followed by DrSmile^®^ (34.65%) and SmileDirectClub^®^ (17.82%). Chi-square tests revealed a significant difference in choosing companies for bite correction, with fewer SmileDirectClub^®^ users selecting treatment for this reason (*p* = 0.027).

### 3.4. Consultation with Dental Professionals

A total of 57.43% of respondents did not consult their regular dentist before starting treatment. Among those who did, 72.09% received a recommendation to seek treatment from a specialized orthodontist in a clinic, while only 11.63% received a favorable recommendation towards direct-to-consumer aligners.

### 3.5. Fit and Insurance Usage

The fit of the aligners varied among companies: 70.59% of DrSmile^®^ users, 82.98% of Impress^®^ users, and 83.33% of SmileDirectClub^®^ users reported that their aligners fit properly. Differences among brands were not statistically significant (*p* = 0.353). Regarding insurance availability, 73.27% of respondents indicated that no insurance option was offered. Among those with insurance, 20% of DrSmile^®^ users, 7.69% of Impress^®^ users, and 50% of SmileDirectClub^®^ users had to use their coverage, although these differences did not reach statistical significance (*p* = 0.161).

### 3.6. Discomfort and Complications

Discomfort experienced with aligners significantly varied among companies (*p* = 0.041). Impress^®^ users reported the lowest discomfort levels, whereas 50% of DrSmile^®^ users reported moderate pain. Complications—including gum inflammation (45.45%), difficulty biting correctly (48.48%), and irritation (42.42%)—were reported by 29.79–37.14% of users, depending on the company, with no significant intercompany differences (*p* = 0.782). However, DrSmile^®^ users were significantly more likely to consult their dentist due to complications, compared to SmileDirectClub^®^ users (*p* = 0.047).

### 3.7. Customer Service and Communication

Attempts to contact the companies were very common, but success rates differed notably: Impress^®^ (60% success), DrSmile^®^ (45.45%), and SmileDirectClub^®^ (77.78%). Significant differences in communication methods were observed (*p* = 0.001), with Impress^®^ users preferring phone calls, while DrSmile^®^ and SmileDirectClub^®^ users relied more on online messaging.

### 3.8. Treatment Satisfaction and Recommendation

Overall, 60% of respondents stated that their expectations were not met, primarily due to communication issues, delays, and unsatisfactory dental outcomes. Consequently, 64% would not recommend the treatments, with the highest dissatisfaction noted among SmileDirectClub^®^ users (77.78%). Differences across companies in terms of satisfaction and recommendations were not statistically significant (*p* = 0.301).

### 3.9. Open-Ended Comments and Feedback

Respondents had the opportunity to leave additional comments regarding their experience with direct-to-consumer aligners. Among the primary emerging themes was frustration with customer service, which was mentioned by more than half of the participants. Recurring comments included complaints about delays in aligner delivery and difficulties in obtaining clear responses from the company. On the other hand, some users highlighted the convenience of the treatment, mentioning that it was more accessible compared to traditional orthodontics. However, several respondents reported experiencing worsening bite alignment or dental stability after treatment, raising concerns about the professional supervision of these procedures.

## 4. Discussion

A total of 517 social media profiles were contacted, resulting in 102 responses, of which 101 were valid after excluding one incomplete survey. The response rate was relatively low, raising the possibility of non-response bias, as the individuals who did not participate may have had different experiences. To mitigate this, the survey was distributed across multiple platforms, and invitations were framed neutrally to avoid influencing participation. Additionally, selection bias may have occurred, as participants with strong opinions—either positive or negative—about DTCO may have been more motivated to complete the survey. Despite these limitations, this study represents an important initial exploration of DTCO user experiences in Spain, providing a foundation for future studies with larger and more diverse samples.

### 4.1. Sociodemographic Trends

Indeed, the sociodemographic composition of the sample—predominantly female (74.26%) and largely full-time employed (58.42%)—may represent a potential bias, limiting the generalizability of our findings to the broader Spanish population. This should be acknowledged, emphasizing the need for cautious interpretation and suggesting future studies with more diverse samples. Nevertheless, this demographic profile aligns with those reported in similar studies on adult orthodontic treatments, where higher proportions of female participants and individuals with stable employment or higher educational levels are commonly observed [12,13]. Despite the difference in size, when the sociodemographic data from our study are compared with those in the study of Wexler et al. (2020), both studies reveal a similar sociodemographic profile of direct-to-consumer aligner users [5]. Most participants in both groups were female, with Millennials representing the dominant age group. Education levels were also high in both studies, with most participants holding at least a university degree. In terms of employment, the majority were full-time workers, indicating financial stability among users. These findings suggest that DTC aligners are primarily chosen by educated, working professionals who value affordability and convenience in orthodontic treatment.

### 4.2. Treatment Motivations and Decision Making

Cost was the dominant factor influencing participants’ decisions to undergo DTCO treatment, followed by convenience and time-saving benefits. These findings are in line with previous studies indicating that affordability is the primary reason for choosing DTCO over conventional orthodontics [13,14]. However, while cost is a driving force, it does not necessarily correlate with treatment satisfaction, as 60% of respondents reported that their expectations were not met. This discrepancy highlights the need for clearer patient education regarding the potential limitations of DTCO treatments. Tabbaa et al. (2023) found that mild or moderate cases were significantly more likely to be addressed via DTCO (3.53 and 1.79 times more probable, respectively, compared to complex cases) [15]. While our study did not apply a formal complexity index, such as the American Board of Orthodontics’ Discrepancy Index, we observed that most users sought DTCO for moderate crowding and esthetic concerns, aligning with the perception that these treatments are sufficient for cases deemed “simple”. Similarly, Tuncer et al. (2015) have confirmed that esthetics—particularly crowding—is the primary motivation for seeking orthodontic treatment; this was reflected in our study, where 66.34% of participants cited it as a key factor in their decision making [13].

### 4.3. Complications and Treatment Outcomes

A significant portion of participants (29.79–37.14%, depending on the brand) reported experiencing complications, including gum inflammation, occlusal instability, and orofacial pain. This aligns with previous research documenting similar adverse effects in DTCO users [16,17]. Regarding potential risks or drawbacks, Patil et al. have emphasized the lack of detailed clinical supervision, the possibility that patients may have undiagnosed periodontal disease, or occlusal and functional complications [16]. Additionally, our study revealed that over half of those who faced complications sought professional dental care, reinforcing concerns that DTCO lacks adequate clinical oversight. Our results align with other studies examining DTCO-related complications. Between 53.85% and 76.92% of affected users required professional evaluation to address unresolved issues, emphasizing that many of the patients ultimately needed traditional dental care, despite choosing DTCO. Similarly, Belgal et al. (2023) [17] reported that 40% of adverse events in the MAUDE database involved bite problems, 30% involved orofacial pain, and 26% were related to periodontal conditions. Our study found a similar pattern, with bite instability (~48%), irritation or pain (~42%), and gingival inflammation or recession (~45%) being among the most common complications [17]. Furthermore, Belgal et al. highlighted that 69.2% of patients experiencing adverse events sought consultation with a dentist unaffiliated with the DTCO company, reinforcing concerns about the adequacy of remote treatment models. These findings suggest that, while DTCO may offer affordability and convenience, its limitations in clinical oversight can result in a higher need for post-treatment intervention by traditional dental professionals [17].

### 4.4. The Role of Social Media in DTCO Adoption

Social media was the primary source of information for 64% of participants, underscoring its significant influence in shaping patient decision making. This finding aligns with prior studies on digital healthcare marketing, where targeted advertising and influencer endorsements have played an increasing role in the promotion of medical and dental services [18,19]. More broadly, social media has transformed healthcare marketing through increased patient engagement, improving health literacy, and providing platforms for patient–provider communication [20,21]. However, the unregulated nature of social media marketing raises ethical concerns, as promotional content may omit critical information regarding treatment risks, alternative options, or necessary clinical evaluations.

### 4.5. Ethical and Regulatory Considerations

The rapid expansion of DTCO raises ethical and regulatory questions regarding patient safety, informed consent, and professional oversight. Previous research has highlighted concerns about the lack of transparency in DTCO marketing strategies, including the use of confidentiality clauses to suppress negative reviews [5]. Our findings suggest that inadequate customer service, difficulties in communication, and unmet treatment expectations contribute to dissatisfaction, which may be exacerbated by unrealistic marketing portrayals. Regulatory bodies should consider implementing stricter guidelines to ensure that patients receive accurate information before committing to DTCO treatments.

### 4.6. Study Limitations and Future Directions

Several limitations must be considered when interpreting our findings. This study relied on self-reported survey data, introducing potential biases such as recall bias and self-selection bias. Additionally, the cross-sectional design prevented us from assessing long-term treatment outcomes or satisfaction trends over time. The sample size, while adequate for detecting overall trends, was not large enough to conduct robust subgroup analyses between companies. Future research should aim to address these limitations by incorporating longitudinal studies, objective clinical assessments, and larger and more diverse sample populations.

### 4.7. Practical Implications and Recommendations

Our findings have important implications for orthodontists, policymakers, and patients. Orthodontists should proactively educate patients on the benefits and risks of DTCO, emphasizing the importance of professional supervision. Policymakers should consider introducing stricter regulations on DTCO marketing practices, in order to ensure transparency and patient safety. Finally, patients should be encouraged to seek professional consultations before committing to DTCO treatment, thus minimizing the risk of complications and dissatisfaction.

## 5. Conclusions

Direct-to-Consumer Orthodontics (DTCO) is an emerging treatment option which is gaining in popularity due to its accessibility and affordability; however, notable limitations exist. In most cases, expectations are not met due to communication barriers, treatment concerns, and service quality issues. Many users experience complications, such as bite instability, gingival inflammation, and orofacial pain, often requiring a visit to a dentist for evaluation and corrective treatment. Social media plays a dominant role in the discovery of such treatments, highlighting its growing influence in healthcare marketing. However, the unregulated nature of DTCO advertising raises ethical concerns regarding transparency, informed consent, and patient expectations. The heavy reliance on digital marketing strategies underscores the need for stricter advertising guidelines, thus ensuring that consumers receive accurate and balanced information before committing to treatment. Future research should investigate long-term patient outcomes and assess the broader impacts of digital marketing on patient decision making and treatment satisfaction.

## Figures and Tables

**Table 1:** The 30 questions included in the Google form sent to the possible respondents.

No.	Question	Possible Answers
**Sociodemographic and Personal Data**		
**1**	What is your gender?	Male; Female; Prefer not to answer
**2**	Which generation do you belong to?	Baby Boomer (1949–1968); Generation X (1969–1980); Millennial (1981–1993); Generation Z (1994–2010)
**3**	What is your marital status?	Single; In a relationship; Married; Divorced; Other
**4**	What is your education level?	High school; Higher Vocational Education; University Degree; Master’s Degree
**5**	What is your employment status?	Student; Self-employed; Part-time employee; Full-time employee
**6**	Do you consider yourself a demanding person?	Not much; Average; Very
**Questions about the Choice of Treatment**		
**7**	With which company did you order the aligners?	Impress^®^; DrSmile^®^; Smile Direct Club^®^
**8**	For whom did you select this treatment?	For myself; For my child or a family member aged 13–17; For my child or a family member over 18; Other
**9**	How did you discover this type of treatment?	TV/Internet advertisements; Social Media (Instagram, Facebook, TikTok, etc.); Recommendation from acquaintances; Other
**10**	Why did you decide to undergo treatment?	Dental crowding; Correcting bite alignment; Gaps between teeth; Other reasons
**11**	What were the reasons for choosing this treatment?	Time-saving; Comfort; Cost; Other
**12**	Have you consulted your regular dentist about this type of treatment?	Yes; No
**13**	What did your dentist advise about this treatment?	Advises against DTCO treatment; Recommends DTCO treatment; Recommends treatment with aligners in a clinic through an orthodontic specialist; Recommends treatment with metal braces; Other
**Questions about the Treatment Experience**		
**14**	Did the chosen company offer any type of insurance once the aligners arrived at your home?	Yes; No
**15**	Have you had to use the insurance?	Yes; No
**16**	Do you think the insurance would cover part of the cost?	Yes; No; Not sure
**17**	How would you describe the feeling of wearing aligners?	No discomfort; Mild pain; Moderate pain; Severe pain; Other
**18**	Did the aligners you received fit your mouth?	Yes; No
**19**	Did you experience any complications?	Yes; No
**20**	What complications did you experience?	Cavities; Gum inflammation; Irritation; Gum recession; Difficulty biting properly; Other
**21**	Due to these complications, did you have to consult your regular dentist?	Yes; No
**22**	Did you try to contact the aligner company?	Yes; No
**23**	Were you successful in contacting them?	Yes; No
**24**	By what means did you manage to get in touch?	Phone call; Online message; Both phone and online message; Never managed to get in touch
**25**	Did they offer to assist you personally?	Yes; No
**26**	Did the person from the aligner company who attended you seem sufficiently qualified?	Yes; No; Other
**27**	Is the treatment meeting or did it meet your expectations?	Yes; No
**28**	Could you briefly tell us the reasons why your expectations are or are not being met?	Open question
**29**	Would you recommend this company’s treatment to your family?	Yes; No
**30**	If you want to participate in the gift card giveaway, please let us know how to contact you	Open question

**Table 2:** Sociodemographic characteristics of the study participants, including gender, generation, marital status, education level, employment status, and self-perceived demand for quality. The number of respondents for each answer (N) and the percentage are displayed.

Variable	Category	N	Percentage
**Gender**			
	Male	26	25.74%
	Female	75	74.26%
**Generation**			
	Baby Boomer (1949–1968)	3	2.97%
	Generation X (1969–1980)	15	14.85%
	Millennial (1981–1993)	43	42.57%
	Generation Z (1994–2010)	40	39.60%
**Marital Status**			
	Single	36	35.64%
	In a relationship	35	34.65%
	Married	26	25.74%
	Divorced	4	3.96%
**Education**			
	High School	19	18.81%
	Higher Degree	23	22.77%
	University Degree	26	25.74%
	Master’s Degree	33	32.67%
**Employment Status**			
	Self-Employed	16	15.84%
	Unemployed	3	2.97%
	Student	7	6.93%
	Retired	1	0.99%
	Full-time Employee	59	58.42%
	Part-time Employee	15	14.85%
**Do you consider yourself a demanding person?**			
	A lot	51	50.50%
	Normal	39	38.61%
	A little	11	10.89%

## Data Availability

Data available on request from the authors.
